# Targeted Degradation Technologies Utilizing Autophagy

**DOI:** 10.3390/ijms26146576

**Published:** 2025-07-08

**Authors:** Zeyu Zhou, Jiaming Liang, Binghua Cheng, Yanyan Li, Wenjie Zhou, Hui Tian, Wenli Shi, Ke Liu, Lijing Fang, Hongchang Li, Ximing Shao

**Affiliations:** 1 Department of Biomedical Engineering, Southern University of Science and Technology, Shenzhen 518000, China; zy.zhou1@siat.ac.cn (Z.Z.); wj.zhou@siat.ac.cn (W.Z.); h.tian@siat.ac.cn (H.T.); 2 Faculty of Pharmaceutical Sciences, Shenzhen University of Advanced Technology, Shenzhen 518028, China; ke.liu@siat.ac.cn (K.L.); lj.fang@siat.ac.cn (L.F.); 3 Guangdong Key Laboratory of Nanomedicine, CAS-HK Joint Lab of Biomaterials, Shenzhen Institutes of Advanced Technology, Chinese Academy of Sciences, Shenzhen 518055, China; jm.liang@siat.ac.cn (J.L.); bh.cheng@siat.ac.cn (B.C.); yy.li@siat.ac.cn (Y.L.); wl.shi@siat.ac.cn (W.S.); 4 University of Chinese Academy of Sciences, Beijing 100049, China

**Keywords:** targeted protein degradation, autophagy, lysosome, drug discovery

## Abstract

Targeted degradation technologies, primarily referring to targeted protein degradation, have emerged as promising drug discovery strategies. In contrast to traditional “occupancy-driven” inhibition approaches, these technologies ingeniously leverage the cell’s endogenous degradation mechanisms to achieve specific elimination of disease-causing targets. Autophagy, a highly conserved cellular clearance pathway, possesses broad substrate recognition capabilities, enabling degradation of not only individual proteins but also protein aggregates, damaged organelles, and invading pathogens. Given these characteristics, researchers are actively exploring the application of autophagy mechanisms in targeted degradation technologies. Herein, we summarize recent advances in autophagy-dependent degradation approaches, including autophagosome tethering compounds (ATTEC), autophagy-targeting chimeras (AUTAC), autophagy-targeting Chimera (AUTOTAC), chaperone-mediated autophagy (CMA)-based methods, nanotechnology-based strategies, and the newly introduced autophagy-induced antibody (AUTAB) technique, highlighting their mechanisms, advantages, and potential applications in treating tumors, neurodegenerative diseases, and other challenging conditions.

## 1. Introduction

Traditional small molecule inhibitors work through an “occupancy-driven” pharmacological mode, which requires high-affinity compounds to bind to the active sites of proteins continuously [1]. However, many proteins do not have a clearly defined active site that inhibitors can bind. This results in traditional small molecule inhibitors covering less than 20% of known proteins with therapeutic potential [2]. Most unaddressed disease targets, such as transcription factors, scaffold proteins, misfolded proteins and other non-enzymatic proteins, are undruggable [3]. There are several reasons for this, including (1) the absence of ligand-binding pockets on target proteins, (2) the reliance on protein–protein interactions for their function, and (3) the variable tertiary structure of the target proteins [4]. The emergence of the targeted degradation technology has overcome the limitations of traditional inhibitors and provides a new way to solve the issue of undruggable protein.

Targeted degradation technology is a precise and efficient biotechnology method. Its core lies in using endogenous pathways to degrade target proteins rather than just inhibiting their function [5]. For example, this technology can be used to target and degrade overexpressed or abnormal proteins in tumor cells, such as specific oncogene products, thereby effectively inhibiting the growth and metastasis of tumors. Targeted degradation has emerged over the past two decades, and many technologies have been developed. According to the degradation pathway utilized, they can be subdivided into two categories: the ubiquitin-proteasome pathway and the lysosomal pathway [6].

Proteasome-targeted chimaeras (PROTACs) are the most representative technology employing the ubiquitin-proteasome pathway [7]. Compared with traditional inhibitors, PROTACs have the advantages of a broader target range, low dosage, low toxicity, and high selectivity. Currently, various PROTAC drugs have entered the clinical research stage [8], among which ARV-471 and ARV-110, which target the estrogen receptor (ER) and androgen receptor (AR), have entered phase III and phase II clinical trials, respectively. In addition, other PROTAC drugs are in various stages of clinical research, targeting targets such as Interleukin-1 receptor-associated kinase 4 (IRAK4), Signal Transducer And Activator Of Transcription 3 (STAT3), Bruton’s tyrosine kinase (BTK), Tropomyosin receptor kinase (TRK), and Bromodomain-containing protein 9 (BRD9). Lysosome-targeted chimaeras (LYTACs) are the targeted degradation technology harnessing the endosome-lysosome pathway [9]. LYTACs can degrade extracellular and cell membrane proteins, which makes up for some of the shortcomings of PROTACs. However, there are still some problems with PROTACs and LYTACs technologies. For example, PROTAC technology relies on the proteasome [10], which is inefficient when degrading bulk protein aggregates. Additionally, some diseases are proteasome-resistant or produce cytotoxic products during proteasome degradation. The effectiveness of LYTACs, on the other hand, hinges on receptors located on the cell membrane [11]. The endocytosis efficiency of LYTACs depends on the expression level of these cell surface receptors, which is a challenge to the universality of the LYTACs technology.

Consequently, more and more researches are turning to the autophagy-lysosome pathway. The autophagy-lysosome pathway is an important intracellular degradation pathway [12]. Autophagy-lysosome degradation has many advantages: (1) it can target not only individual proteins, but also protein aggregates and organelles; (2) it does not rely on ubiquitination, avoiding the limitations of the ubiquitination pathway, such as insufficient expression of E3 ligases or limited proteasome function; (3) it has advantages in processing target proteins that are resistant to PROTACs, and can process intracellular proteins that are not suitable for LYTACs. The autophagy-lysosomal degradation technology has significant advantages in terms of degradation range, selectivity, applicable disease types and potential safety, and this technology shows unique potential in processing neurodegenerative diseases induced by bulk protein aggregates. Therefore, this review summarizes the mechanisms and characteristics of current autophagy-based degradation strategies, including ATTEC, AUTAC, AUTOTAC, CMA pathway-based techniques, and our recently developed AUTAB, in order to provide a reference for the further development of autophagy-mediated targeted degradation techniques.

## 2. The Concept and Basic Principles of Autophagy

**Figure 1:**
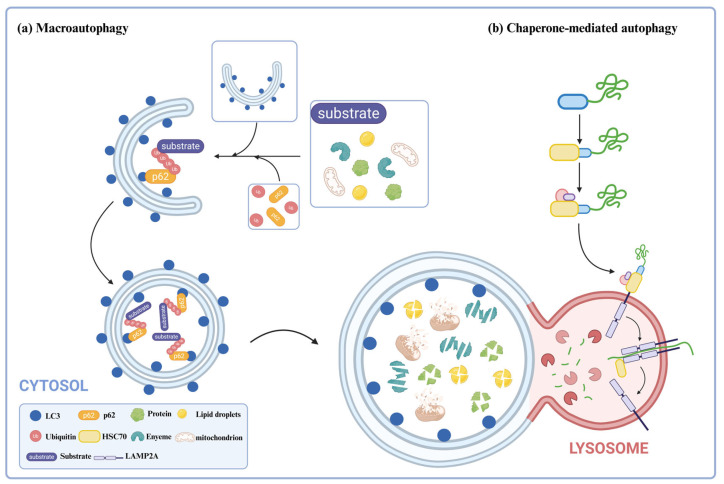
The mechanism of macroautophagy and chaperone-mediated autophagy. (**a**) Macroautophagy involves forming a double-membrane autophagosome that engulfs cytoplasmic substrates, with LC3-II stabilizing membrane expansion. The autophagosome fuses with lysosomes to form autolysosomes, where hydrolases degrade substrates for recycling. (**b**) Chaperone-mediated autophagy involves HSC70 recognizing proteins with KFERQ motifs, which bind to LAMP2A on the lysosomal membrane. LAMP2A oligomerizes to form a translocation complex; HSC70 aids in the unfolding and translocation of substrates into the lysosome for degradation.

Autophagy is a process in which eukaryotic cells degrade their cytoplasmic proteins and damaged organelles using lysosomes under the regulation of autophagy-related genes [13]. It plays a role in maintaining the stability of the intracellular environment. Autophagy activity is assessed by autophagic flux [14]. Autophagic flux refers to the intensity of the autophagic process that occurs within a certain period, mainly related to the intensity of autophagy induction and the rate of autophagosome fusion and digestion by lysosomes. Autophagy can be divided into three types: macroautophagy [15], microautophagy [16], and chaperone-mediated autophagy [17]. This review focuses on introducing the types of autophagy utilized in current degradation technologies, specifically macroautophagy (hereafter referred to as “autophagy”) and chaperone-mediated autophagy (Figure 1).

Autophagy is one of the most widely studied biological processes. Under normal conditions, the level of autophagy in cells is low, but it is activated under stress conditions such as starvation, hypoxia, and oxidative stress [18]. When activated, a double-membrane structure, called phagophore, arises from the endoplasmic reticulum and progressively engulfs the cytoplasmic material, and after its sealing, forms the autophagosome. The autophagosome then fuses with the lysosome to form an autophagolysosome, and the hydrolases in the lysosome finally degrade the cytoplasmic material [19,20]. The resulting degradation products can be reused by the cell. During autophagy, autophagosome formation and maturation are regulated by various autophagy-related proteins (ATG) [21]. For example, ATG8/MAP1LC3 (microtubule-associated protein one light chain 3, or LC3 for short) is essential for the formation (initiation, extension, and closure) and maturation of autophagosomes. LC3 is the mammalian homologue of ATG8 [22,23]. The C-terminus of ATG8/LC3 is hydrolyzed by the ATG4 cysteine protease, resulting in the formation of an exposed glycine residue, generating LC3-I, which is located in the cytoplasm [24]. The exposed glycine residue forms a thioester bond under the action of the E1 enzyme ATG7. The E2 enzyme ATG3 binds to LC3-I via the thioester bond; the C-terminal glycine residue of the cytosolic LC3-I is coupled to phosphatidylethanolamine (PE) by the ATG12-ATG5-ATG16 complex to form LC3-PE (hereinafter referred to as LC3-II); LC3-II is then attached to the membrane of the autophagosome being formed [25]. During degradation, LC3-II on the outer membrane of the autophagosome is cleaved by ATG4 to form LC3-I, which is reused, while LC3-II on the inner membrane is degraded together with cytoplasmic contents [26]. LC3 is currently used as a specific marker protein for autophagy.

Autophagy is a process of non-selective degradation of intracellular material, but studies have shown that autophagy can also be highly selective [27]. Here, p62/SQSTM1 was the identified selective autophagy receptor, and its function has been linked to various diseases, including neurodegenerative diseases and cancer [28]. It acts as a bridge between LC3 and the ubiquitinated proteins engulfed by the growing autophagosome [29]. LC3 family proteins have binding sites for autophagy receptors, namely, LDS (LIR-docking site) for binding to the LIR sequence on the autophagy receptor. p62/SQSTM1 consists of three domains: the PB1 domain at the N-terminus for self-aggregation, the LIR domain for interaction with LC3, and the UBA domain for interaction with ubiquitinated proteins. Under physiological conditions, the UBA domain of p62/SQSTM1 binds to ubiquitinated proteins, which are then selectively captured into autophagosomes through the interaction of LIR and LC3. After the formation of autophagolysosomes, ubiquitinated proteins are degraded. Therefore, changes in the level of p62/SQSTM1 can also be used to reflect the status of the autophagy flux. When LC3-II is elevated, and p62/SQSTM1 is reduced, this indicates that the autophagic flow is unobstructed; when LC3-II is elevated, and p62/SQSTM1 is elevated, this indicates that autophagy initiation is normal, but the downstream is blocked, and the autophagosomes and lysosomes cannot fuse.

CMA is a unique protein degradation pathway, in which cytoplasmic proteins selectively degrade by passing directly through the lysosomal membrane without forming additional vesicles [30]. During the process of CMA, molecular chaperones, such as heat shock protein 70 (HSC70), recognize substrate proteins that carry a specific amino acid sequence in the cytoplasm [31]. The specific amino acid sequence is a pentapeptide that has been identified as Lys-Phe-Glu-Arg-Gln (KFERQ), and it has been shown that combinations of pentapeptides with similar properties to this amino acid sequence can be recognized by HSC70 [30]. HSC70 recognizes and binds to substrate proteins, triggering lysosomal targeting. Upon reaching the lysosome, the molecular chaperone-substrate complex binds to the receptor lysosome-associated membrane protein type 2A (LAMP2A), and the substrate is unfolded and forms a CMA translocation complex, with HSC70 in the lysosome mediating the translocation of the substrate. As a result, the substrate will be degraded by hydrolases when it enters into the lysosome.

## 3. Autophagic Degradation Strategy Using LC3-Binding Compound

**Figure 2:**
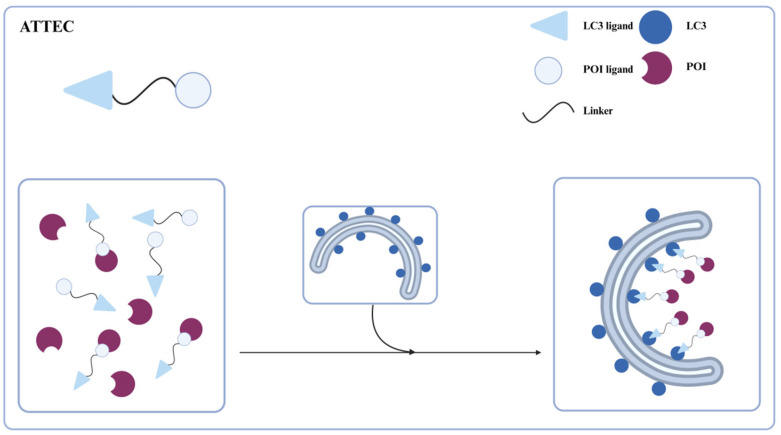
Schematic representation of ATTEC for protein degradation. ATTECs concurrently bind to both the POI and LC3 proteins. This LC3 engagement facilitates the engulfment of the POI by forming autophagosomes, ultimately leading to POI degradation in the autolysosome.

Li et al. in 2019 [32] reported a novel, direct targeted protein degradation strategy, ATTECs, for degrading certain large proteins or aggregates via the autophagy-lysosomal pathway (Figure 2). ATTECs are phagophore-binding compounds consisting of a linker and two ligands that bind to the protein of interest (POI) and LC3, respectively. The binding of LC3 facilitates the POI engulfment into the growing autophagosome, leading to the degradation of POI in the autolysosome. Bypassing the ubiquitination of substrates, ATTECs technology not only allows for the selective degradation of large proteins or aggregates, but also of a wide range of biological macromolecules, lipids, and even organelles. Moreover, ATTEC molecules exhibit relatively lower molecular weights, thereby enhancing their therapeutic potential and clinical translatability. Next, we will provide a detailed introduction to the different applications of ATTECs.

### 3.1. ATTECs in Protein Degradation

**Figure 3:**
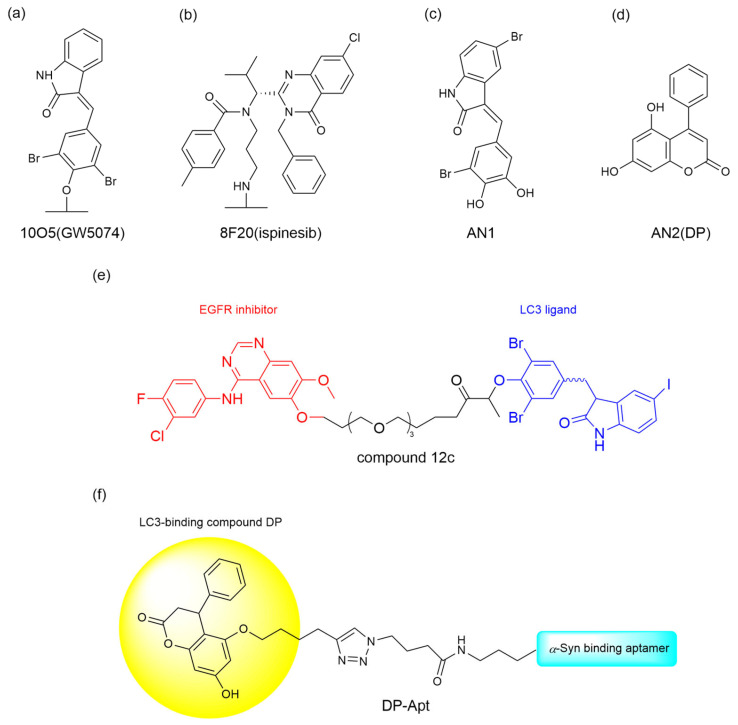
The chemical structures of ATTEC for protein degradation. (**a**–**d**) These compounds have affinity for LC3 and mHTT. (**e**) Compound 12c targeting EGFR. (**f**) DP-Apt targeting α-Syn.

Mutant Huntington protein (mHTT), a large 350 kDa protein, forming abnormal large molecular clusters that accumulate in the brain and damage the function of nerve cells, causes Huntington’s disease (HD) and neurodegeneration [33]. To degrade the mutant huntingtin (mHTT) in Huntington’s disease, Li et al. [32] identified four promising ATTEC candidates—10O5 (GW5074), 8F20 (ispinesib), AN1, and AN2—through small molecule microarray screening of 3375 compounds. These ATTECs exhibited selective binding affinity for LC3B and mHTT while sparing wild-type HTT (Figure 3a–d). Subsequent dose-response experiments demonstrated these compounds’ efficacy in mHTT degradation, with autophagy inhibitors NH4Cl and chloroquine further validating the autophagic mechanism underlying this therapeutic effect. The team further investigated the effect of these compounds in vivo. In experiments on both HD transgenic fruit fly models and HD knock-in mouse models, all four compounds reduced mHTT levels in a significant allele-selective manner, while no increase in mHTT aggregates was observed. The successful degradation of mHTT suggests a promising potential for treating HD in patients.

Epidermal growth factor receptor (EGFR), a tyrosine kinase receptor regulating cell proliferation, survival, and migration, is frequently mutated or overexpressed in various tumors, especially in non-small cell lung cancer (NSCLC), driving tumor progression and therapy resistance [34,35]. Although EGFR tyrosine kinase inhibitors (TKIs) are clinically used to treat NSCLC, their acquired resistance driven by EGFR mutations remains a major driver of malignant progression [35]. To overcome this obstacle, Zhu et al. [36] developed EGFR-ATTEC molecules that degrade intracellular EGFR, by linking the LC3 ligand GW5074 and first generation TKIs with different linkers to address the acquired drug resistance of EGFR mutants. The EGFR-ATTECs were evaluated for their anti-proliferative activity in a panel of cell lines bearing different EGFR mutations. While first-generation TKIs selectively target EGFR-Del19 and L858R mutations, EGFR-ATTECs exhibit potent anti-proliferative effects in cell lines harboring these oncogenic variants; however, they show no activity against other EGFR models like A549 EGFR-WT or H1975 EGFR-L858R/T790M. Given that, compound 12c (Figure 3e) was found to have the best dose- and time-dependent degradation effect in HCC827 EGFR-Del19, and mechanistic studies confirm its autophagy-lysosomal pathway dependency via autophagy inhibitor or enhancer modulation. In vivo studies revealed dose-dependent tumor suppression and rapid EGFR degradation (24 h) by compound 12c in PC-9 xenografts, alongside favorable safety profiles.

Pathological protein α-synuclein (α-Syn) can form aggregates that contribute to the development of Parkinson’s disease (PD). It has been reported that autophagy enhancers are capable of protecting neurons by promoting the autophagy-lysosomal degradation of α-Syn. In light of this, Liao et al. [37] proposed the use of ATTEC to offer a therapeutic option for α-Syn targeted clearance. They chose a reported α-Syn aptamer (Apt1) with specificity and high affinity and the LC3-binding compound DP to generate the aptamer-ATTEC chimera DP-Apt (Figure 3f). DP-Apt exhibits its ability to reduce the level of α-Syn, both α-Syn monomers and aggregated forms, as well as endogenous α-Syn. The aptamer-ATTEC technology developed by Liao et al. may open a new window for ATTEC-based targeted protein degradation technology.

In addition to small molecule-based or aptamer-based ATTEC technologies, He et al. [38] developed a non-small molecule-based approach using nanobody chimeras (Autophagy-targeting nanobody chimeras, ATNC) for selective degradation of intracellular targets. The ATNC chimera consists of an LIR sequence DSEDEDFEILSL derived from the ATG4B protein that binds to LC3 and a nanobody that binds to the POI. ATNC allows for the autophagic degradation of various tag-fused proteins (GFP-tag, ALFA-tag, BC2-tag) and endogenous targets (Ras Homolog Family Member A(RhoA), Protease HtpX homolog 2 (hTPX2), and then they design GBP-RBP-LC3B ATNC that facilitates the simultaneous degradation of dual tag targets, which is important for developing drugs with synergistic effects. This general, modular, and versatile technology also enables the degradation of other substrates, including organelles and aggregated proteins, broadening the scope of autophagy-based degradation technologies.

### 3.2. ATTECs in Enzyme Degradation

**Figure 4:**
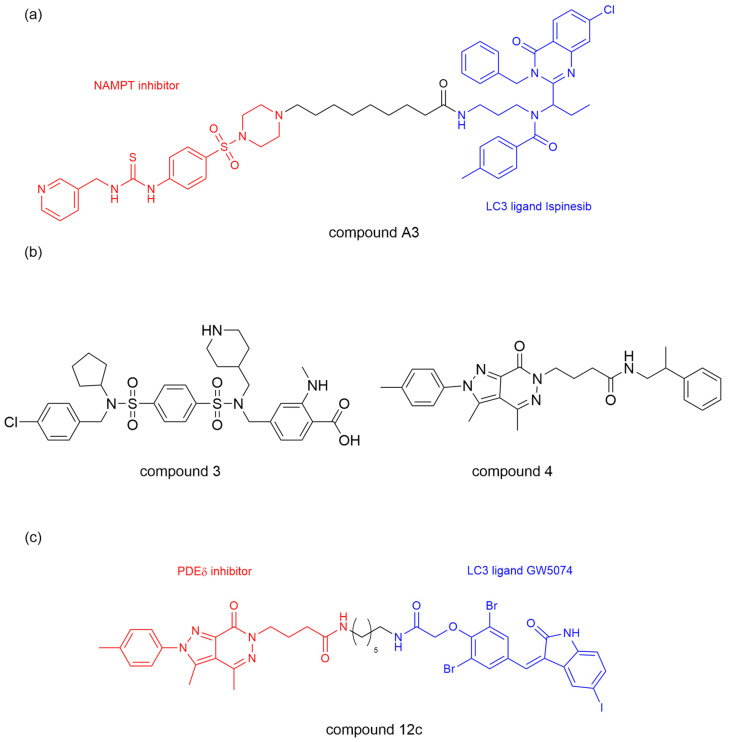
The chemical structures of ATTEC for enzyme degradation. (**a**) Compound A3 targeting NAMPT. (**b**) Compound 3 and 4 have high for affinity PDEδ. (**c**) Compound 12c targeting PDEδ.

Nicotinamide phosphoribosyltransferase (NAMPT) is the rate-limiting enzyme for nicotinamide adenine dinucleotide (NAD) and has been reported to play a critical role in tumor cell proliferation as a direct target of the BRAF oncogenic signaling pathway [39]. Previous efforts to block the NAMPT activity were limited to NAMPT inhibitors FK866 and CHS828 with limited efficacy and dose-dependent toxicity [40]. Dong et al. [41] developed an ATTEC molecule, by linking ispinesib as a LC3 ligand and compound MS2 as a potent NAMPT inhibitor, with different lengths of alkyl or polyethylene glycol. They showed that prolonging the linker length significantly enhanced NAMPT degradation efficiency in A2780 human ovarian cancer cells with peak efficacy (91% at 3 μM) observed with a linker length of *n* = 8 (compound A3, Figure 4a). Moreover, compound A3 showed excellent anti-proliferative activity against multiple cell lines (including A2780, MBA-MB-231, HCT116, and A549), with more significant effects and a nanomolar range IC50 value in NAMPT-overexpressing cell lines. Mechanistically, compound A3 induced NAMPT degradation through the lysosomal-mediated autophagy pathway, which is confirmed by the treatment of various autophagy inhibitors (NH4Cl and chloroquine) that prevented NAMPT degradation. This work verified the feasibility of using ispinesib as a LC3 ligand and successfully developed a NAMPT degrader with anti-tumor activity.

Based on the above research, Bao et al. [42] further studied ATTEC molecules targeting phosphodiesterasesδ (PDEδ). PDEδ critically regulates oncogenic KRAS membrane localization and signal transduction, with KRAS mutations driving ~30% of lung, 45% of colorectal, and 90% of pancreatic cancers. Targeting the KRAS–PDEδ interaction shows therapeutic potential, though current inhibitors suffer from poor in vivo efficacy [43,44,45]. The team leveraged LC3-binding compounds 1 (GW5074) and 2 (ispinesib), previously validated in BRD4 and NAMPT degradation, alongside compounds 3-4, high-affinity PDEδ inhibitors, to synthesize several PDEδ ATTEC compounds (Figure 4b) [46,47]. Compound 12c emerged as the optimal PDEδ ATTEC, achieving 85% degradation in KRAS-mutant MiaPaCa-2 cells (Figure 4c). It exhibited concentration- and time-dependent PDEδ degradation with concurrent apoptosis induction, demonstrating enhanced anti-proliferative activity (IC50 = 1.4 μM in MiaPaCa-2; 0.8 μM in Capan-1), outperforming the PDEδ inhibitor (compound 4) by >10-fold. Mechanistic studies revealed LC3-dependent PDEδ degradation by compound 12c via the autophagy-lysosomal pathway, as evidenced by Bafilomycin A1 blockade. This degradation occurred without altering PDEδ mRNA levels as confirmed by qRT-PCR. PDEδ ATTEC also has an influence on downregulating the expression level of p-AKT, suggesting that the downstream signaling pathways of KRAS could be improved indirectly. This study further validates the effectiveness of ATTEC in TPD technology, expands the scope of degradable targets, and provides promising lead compounds for treating KRAS–PDEδ interactions.

### 3.3. ATTECs in Lipid Droplets Degradation

Liquid droplets (LDs), the cell’s lipid storage structure, consists of a hydrophobic core of neutral lipids enclosed by a phospholipid monolayer membrane that is decorated by a specific set of proteins [48]. Abnormal accumulation of LDs is associated with many diseases, such as obesity, cardiovascular disease, fatty liver, and neurodegenerative diseases [49,50,51]. To clear the non-protein pathogenic molecules, such as LDs, Fu et al. [52] hypothesized that ATTECs interacting with both LC3 and LDs may induce the autophagic degradation of LDs.

**Figure 5:**
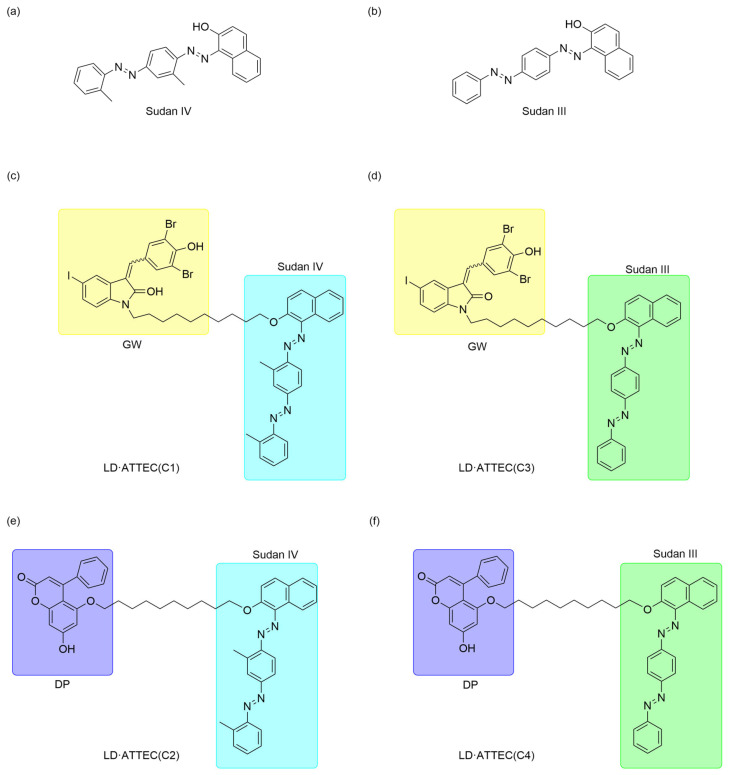
The chemical structures of ATTEC for liquid droplet degradation. (**a**) The structure of Sudan IV. (**b**) The structure of the Sudan III. (**c**–**f**) LD·ATTEC targeting liquid droplets.

Therefore, they designed LD·ATTECs by linking the LC3 ligands GW5074 and DP to the lipid droplet (LD)-binding detection probes Sudan IV (SIV) (Figure 5a) and Sudan III (SIII) (Figure 5b), respectively, synthesizing four LD·ATTECs designated C1-C4 (Figure 5c–f). LD·ATTECs bridge lipid droplets (LDs) and LC3B, assembling an LD-LD·ATTEC-LC3B ternary complex that triggers autophagosome engulfment, thereby delivering lipid cargo to lysosomes for degradation. LD·ATTECs lower the oleic acid (OA)-induced LDs level in wild-type (WT) mouse MEF cells. This strategy showed that LD·ATTECs significantly reduced the number and size of lipid droplets, and this LD-lowering effect was dependent on autophagy, as the degradation was prevented in Atg5-knockout cells. In addition, LD·ATTECs do not affect overall autophagy activity but specifically promote the targeted degradation of lipid droplets.

Most notably, LD·ATTECs show great efficacy in two mouse models with different metabolic disorder conditions. The team chose C3 and C4 for in vivo injection because of their lower molecular weight and higher affinity for LC3B. After intraperitoneal injection of LD·ATTECs, the lipid-lowering effect included weight loss, reduced liver lipid content, and alleviated fibrosis. Fu et al. [52] also discussed the safety of LD·ATTECs and potential directions for optimization, pointing out that Sudan dyes may pose a carcinogenic risk. However, the current experimental dose is low, and they can be replaced with safer lipid droplet probes in the future.

In addition to metabolic disorders, the LD·ATTECs also hold potential for the treatment of hereditary spastic paraplegia (HSP), a group of inherited neurodegenerative diseases defined by progressive lower limb spasticity and weakness [53,54]. Notably, HSP subtype SPG54, caused by mutation of the *DDHD2* gene, was widely speculated and LDs are accumulated in the brains of SPG54 patients [55]. Jia and Wang et al. [53] first discovered the interaction between *DDHD2* and LC3/GABARAPs, wherein *DDHD2* deficiency leads to lipid droplet accumulation, while overexpression of *DDHD2* reduces lipid droplet formation. This mechanistic elucidation provided the theoretical foundation for applying LD-ATTEC to HSP therapy. As hypothesized, LD·ATTEC was shown to effectively lower LDs in OA-induced *DDHD2*-KO HeLa cells or neuronal HT22 cells. Jia et al. [53] applied the ATTEC technology to the degradation of lipid droplets, achieving the targeted degradation of non-protein biomolecules for the first time. This breakthrough in targeted degradation technology from proteins to non-protein substances has opened up new avenues for drug research and development.

### 3.4. ATTECs in Organelle Degradation

Mitochondrial dysfunction or impaired mitophagy plays a key role in many neurodegenerative diseases, such as Parkinson’s disease (PD) and Down syndrome (DS) [56]. Therefore, clearance of damaged mitochondria to restore the mitochondrial homeostasis may provide a promising new strategy for addressing these diseases.

**Figure 6:**
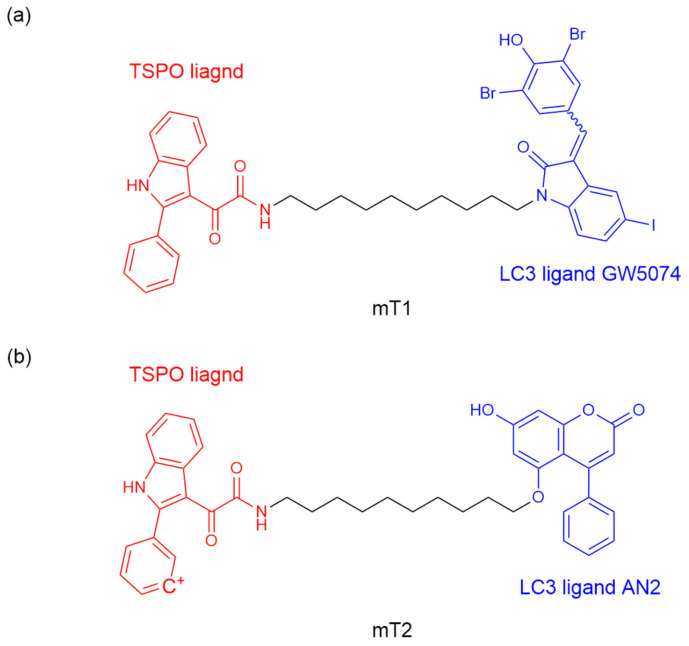
The chemical structures of ATTEC for organelle degradation. (**a**,**b**) mT1 and mT2 targeting damaged mitochondria.

After achieving the degradation of proteins and lipids, Tan et al. [56] explored the strategy of inducing mitochondrial degradation through mitochondrial-targeted autophagy by ATTEC. They designed ATTEC molecules mT1 and mT2 by binding LC3B ligand GW5074, AN2, and 2-phenylindole derivative (a known mitochondrial outer membrane protein TSPO ligand) through a ten-atom carbon chain (Figure 6a,b). ATTEC mT1 but not mT2, shows its capacity of simultaneously tethering LC3B to TSPO, enhancing the engulfment of damaged mitochondria by autophagosomes and subsequent autophagic degradation. Since defects in mitochondrial autophagy are a significant factor in the pathogenesis of neurodegenerative diseases (NDs), the team continued to investigate the potential rescue effect of mT1 on ND-related phenotypes by promoting mitophagy. A PD-related cell model was established using MPP+ treated human neuroblastoma cells (SH-SY5Y), a treatment that causes PD-like pathology. mT1 inhibited the apoptosis induced by MPP+ and potentially rescued the PD-related pathology in PD cell models by promoting mitochondrial degradation.

In addition, the team investigated the potential rescue effect of mT1 on DS-related phenotypes. Dysfunctional mitochondria are abnormally aggregated in induced pluripotent stem cells (iPSCs)-derived neurons and organoids from DS patients. As expected, cortical neurons and cerebral organoids treated with mT1 had reduced mitochondrial aggregation. These results indicated that mT1 can correct the abnormal distribution, morphology, and neuronal loss of mitochondria in DS cortical neurons and brain organoids, suggesting that mT1 has a potential rescue effect on DS-related mitochondrial defects. In summary, Tan et al. [56] demonstrated the potential of ATTEC molecules to enhance mitochondrial autophagy and the effect of using mitochondria-targeted mT1 on ND-related phenotypes, providing potential new strategies for treating ND or related diseases.

## 4. Autophagic Degradation Strategy Based on S-Guanylation Tags

**Figure 7:**
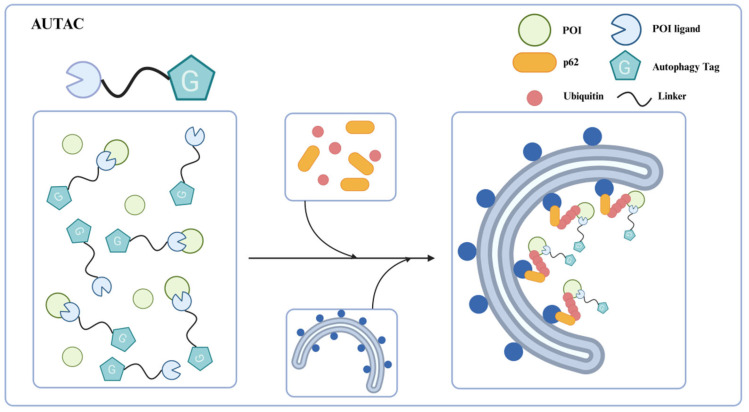
Schematic representation of AUTAC for protein degradation. AUTACs induce K63-linked polyubiquitination of the target protein via its autophagy tag, and then the ubiquitinated protein is recognized by the autophagy cargo receptor p62/SQSTM1, subsequently recruiting the autophagosomes to facilitate lysosomal degradation of the POI.

Xenophagy (antibacterial autophagy) is a suitable model for studying the mechanism of selective autophagy [57]. The group A streptococci (GAS) of invading pathogens are sequestered by GAS-containing autophagosome-like vacuoles (GcAVs) after polyubiquitination and then removal via autophagy-lysosomal pathway [58]. In previous research, Hirokazu Arimoto’s team found that the cGMP modification (S-guanylation) might be responsible for subsequent K63-linked polyubiquitination of the invading GAS [59]. S-guanylation seems to be an important autophagy clearance signal, involved in bacteria recognition and recruitment to the autophagosomes. Based on this discovery, the research team proposed the concept of autophagy-targeting chimeras (AUTACs) in 2019 (Figure 7) [57].

### 4.1. First-Generation AUTAC

The structural design of first-generation AUTAC consists of three parts: a specific ligand that can recognize the intracellular POI, a linker, and a guanine tag that mimics the S-guanylation modification for the polyubiquitination of POI. In contrast to PROTACs hijacking the E3 ligases to catalyze K48-linked polyubiquitination to proteasome degradation, AUTAC induces the K63-linked polyubiquitination of the POI through the autophagy tag. Then, the ubiquitinated protein is recognized by the autophagy cargo receptor p62/SQSTM1 and subsequently recruits autophagosomes for degradation via the lysosomal pathway. The selective recognition by the autophagy receptor p62/SQSTM1 bridges K63-linked polyubiquitination with autophagic degradation, thereby addressing the therapeutic limitations of PROTAC technology.

**Figure 8:**
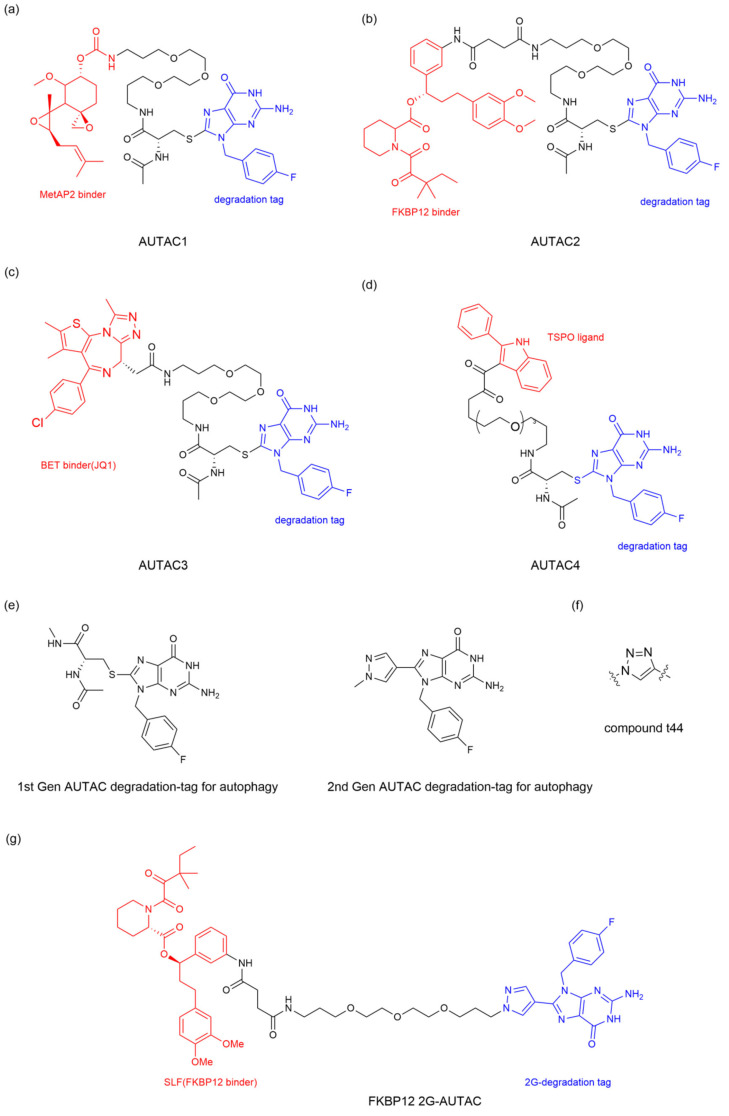
The chemical structures of AUTAC for protein degradation. (**a**) AUTAC1 targeting MetAP2. (**b**) AUTAC2 targeting FKB12. (**c**) AUTAC3 targeting BET. (**d**) AUTAC4 targeting BRD4. (**e**) The structure of degradation tag of 1G-AUTAC. (**f**) The structure of degradation tag of 1G-AUTAC. (**g**) FKBP12 2G-AUTAC targeting FKBP12.

Daiki Takahashi et al. [57] reveal S-guanylation as a standalone tag driving cargo-selective autophagy in the cGMP-HaloTag ligand (cGMP-HTL) mimetic system. Building on this discovery, the team optimized a new ligand lacking the cyclic phosphate moiety: p-fluorobenzyl guanine (FBnG) and designed various AUTAC molecules, effectively avoiding the undesirable off-targeted activation of protein kinase G (PKG) owing to the cGMP substructure. AUTAC1-3 were armed with different POI ligands to expand the scope of targeted degradation (Figure 8a–c). As expected, AUTAC1 and 2, respectively degraded MetAP2, and FKB12 attained 80% clearance of these cytoplasmic proteins. In contrast, AUTAC3 showed limited efficacy of reducing the nuclear protein BRD4. This limited efficacy may be attributed to the fact that canonical autophagy is mainly active in the cytoplasm and not in the nucleus. In addition, the team further investigated the effect of S-guanylation on mitophagy and designed the AUTAC4 molecule using the 2-phenylindole derivative as a mitochondria-targeting binder (Figure 8d). Upon treatment with AUTAC4 and the mitochondrial toxins CCCP, the damaged mitochondria were removed via LC3B-mediated mitophagy; the loss of mitochondrial membrane potential and apoptosis induced by CCCP treatment were attenuated and the energy production was restored by improving mitochondrial quality. As mentioned above, mitochondrial dysfunction is a key contributor to Down syndrome (DS). To clarify the potential therapeutic effect in DS patients, the team applied AUTAC4 to a DS patient’s fibroblasts (Detroit 532 cells) to mediate mitophagy for dysfunctional mitochondria removal. The results showed that the AUTAC4 can induce mitophagy in Detroit 532 cells, and the recruitment of the autophagy marker LC3B can be observed. Collectively, these studies demonstrated that AUTAC as a platform technology is capable of broad application.

### 4.2. Second-Generation AUTAC

The research on the first-generation AUTAC (1G-AUTAC) focused on optimizing the phosphorylated ribose structures found in S-guanylation to avoid the undesirable activation by PKG. In order to optimize the degradation ability of 1G-AUTAC, Hirokazu Arimoto’s team modified 1G-AUTAC through a structure−activity relationship (SAR) research to obtain the second-generation AUTAC (2G-AUTAC, Figure 8e), which showed better degradation efficacy and addressed the issue of the negative charge of cyclic phosphates in the 1G-AUTAC affecting cell membrane permeability [60].

To establish a robust AUTAC detection system, Daiki Takahashi et al. [60] fused the enhanced green fluorescent protein (EGFP) to the HaloTag ligand (HTL) mimetic system as introduced above, named the structural−activity relationship (SAR) study. Hence, high-throughput fluorescence-based analysis could be used to evaluate the robustness of AUTAC degradation induced by test compounds in living cells. For the design of 2G-AUTAC, Daiki Takahashi et al. [60] persistently experimented with a guanine alternative, focusing on other purines or pyrimidines, other positions of the fluorine in the benzyl substituent and other substructures that might replace L-cysteine (L-Cys). They confirmed that the guanine structure is important for the degradation of AUTAC. Substituting the L-Cys residue between the linker and the guanine structure with a benzene ring, pyrazole ring, and triazole ring can effectively improve the degradation of AUTAC, among which the pyrazole ring candidate (compound tt44) demonstrated a remarkable removal effect. Furthermore, they verified that the polyethylene glycol (PEG) length (*n* = 5) is the appropriate linker length for tt44-based AUTAC (Figure 8f). The team designed the FKBP12 2G-AUTAC molecule using the FKBP12 protein-ligand to study the autophagic degradation of FKBP12. The results showed that the FKBP12 2G-AUTAC (Figure 8g) molecule could significantly degrade the FKBP12 protein at a concentration of 1 μM, which was 100 times more efficient than 1G-AUTAC. Further studies on the mechanism found that the degradation of FKBP12 depended on the selective autophagy pathway mediated by p62, consistent with the autophagy-dependent mechanism of 1G-AUTAC.

## 5. Autophagic Degradation Strategy Harnessing p62 Ligand

**Figure 9:**
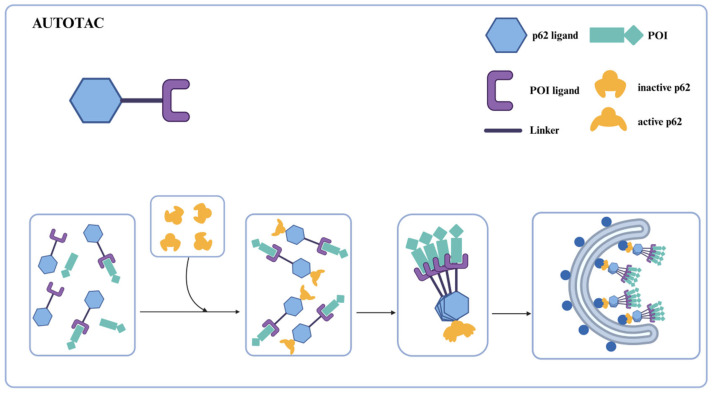
Schematic representation of AUTOTAC for protein degradation. AUTOTACs use dual ligands to bind target proteins and p62/SQSTM1, forming a ternary complex that triggers p62 oligomerization and exposure of the LC3-binding domain. This domain recruits LC3, promoting autophagosome encapsulation, leading to degradation via the autophagy pathway.

**Figure 10:**
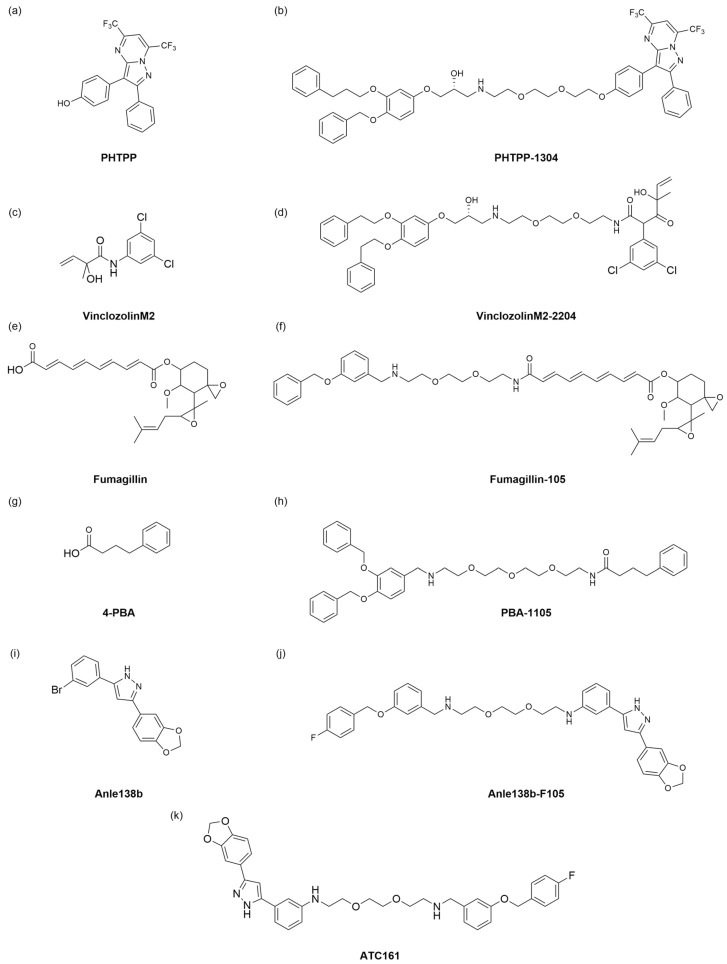
The chemical structures of AUTOTAC for protein degradation. (**a**) The structure of PHTPP. (**b**) PHTPP targeting ERβ. (**c**) The structure of vinclozolinM2. (**d**) VinclozolinM2-2204 targeting AR. (**e**) The structure of Fumagillin. (**f**) Fumagillin-105 targeting MetAP2. (**g**) The structure of 4-PBA. (**h**) PBA-1105 targeting aggregation-prone P301L Tau mutant. (**i**) The structure of Anle138b. (**j**) Anle138b-F105 targeting aggregation-prone P301L Tau mutant. (**k**) The structure of ATC161, the compound targeting α-Syn aggregates.

The Yong Tae Kwon team proposed the AUTOTAC technology (Figure 9) [61]. AUTOTAC is a dual-function molecule with the selective autophagy receptor p62 at its core. AUTOTAC can, in principle, target any given protein for target autophagy degradation and does not depend on the ubiquitination of the target protein. The AUTOTAC molecule consists of three parts: the ligand for the target protein, the linker, and the ligand for the p62 protein. A multi-step mechanism achieves AUTOTAC-mediated targeted degradation. First, AUTOTAC binds to the target protein and p62 through its target-binding ligand and autophagy-targeting ligand, forming a ternary complex. AUTOTAC causes a conformational change in p62, resulting in self-oligomerization and exposure of the LC3-binding domain, which in turn binds to LC3, mediating the isolation of the substrate into the autophagosome and its degradation via the lysosomal pathway. Finally, AUTOTAC cycles from the lysosome to the cytoplasm to provide sustainable degradation of other targets. Based on this, the team designed AUTOTAC molecules by designing various small molecule compounds that can activate p62 activity. Using the non-steroidal and synthetic ligand of estrogen receptor beta (ERβ), they constructed the AUTOTAC (PHTPP-1304) for ERβ (Figure 10a,b). The results showed that the molecule caused p62 oligomerization and significant ERβ degradation in HEK293, ACHN, and MCF7 cells, causing oligomerization of p62 and significant degradation of ERβ. The team then used vinclozolinM2 (Figure 10c) and fumagillin (Figure 10e) to design AUTOTAC molecules that degrade the androgen receptor (AR) and methionine aminopeptidase 2 (MetAP2). The results showed that vinclozolinM2-2204 (Figure 10d) and fumagillin-105 (Figure 10f) could also cause p62 oligomerization and that the former achieved the degradation of AR in LNCaP cells, while the latter achieved the degradation of MetAP2 in HEK293 and U87-MG cells. The above results verified that AUTOTAC is a degrader targeting intracellular soluble proteins.

To overcome the problem of soluble misfolded proteins not being degraded through the narrow inner diameter of the proteasome, the team attempted to apply AUTOTAC to degrade the misfolded proteins and their aggregates. First, based on the universal feature of misfolded proteins that expose hydrophobic regions, they identified the chemical partner, 4-phenylbutyric acid (4-PBA) (Figure 10g), and selected a phase 1 clinical trial compound Anle138b (Figure 10i) to design two AUTOTAC molecules PBA-1105 (Figure 10h) and Anle138b-F105 (Figure 10j) to degrade the aggregation-prone P301L Tau mutant, achieving a DC_50_ of approximately 3 nM. Next, the team tested whether AUTOTAC was suitable for other neurodegenerative proteins, such as Huntingtin. The results showed that PBA-1105 and Anle138b-F105 (Figure 10j) degraded the mutant Huntington protein Q97 and Q103. In the in vivo experiments, the team used the previously developed hTauP301L-BiFC transgenic mouse to evaluate the therapeutic effect of the AUTOTAC molecule PBA-1105 on Tau aggregates. Their results showed that PBA-1105 significantly degraded Tau aggregates in a dose-dependent manner while not affecting wild-type mice. In addition, PBA-1105 significantly reduced the number and fluorescent signal of Tau aggregates in the mouse cortex and hippocampus CA1 region. AUTOTAC provides a platform for degrading pathological protein aggregates in the brain.

Based on the above research, the Yong Tae Kwon team also applied AUTOTAC to treat Parkinson’s disease (PD) [62]. One of the most important and distinctive features of Parkinson’s disease is the aggregation of misfolded alpha-synuclein (α-Syn) in neurons, followed by the formation of Lewy bodies in the substantia nigra, which ultimately leads to the degeneration of dopaminergic neurons [63]. Jihoon Lee et al. [62] first investigated the relationship between α -Syn aggregation and autophagy. By establishing a Parkinson’s cell model that produces excessive α-Syn aggregates and detecting its autophagy flux, Yong et al. found that α-Syn aggregation causes damage to the autophagy membrane, indicating that excessive α-Syn aggregation inhibits autophagy. Next, the team used the previously constructed AUTOTAC platform to degrade excess aggregated α-Syn. Finally, it confirmed that the ATC161 (Figure 10k) molecule could better promote the oligomerization of p62 and effectively induce the degradation of α-Syn aggregates through autophagy. Finally, the team evaluated the pharmacokinetic characteristics of ATC161 administered orally to mice, and the results showed that ATC161 is metabolically stable and is an orally available drug. The in vivo therapeutic effect of ATC161 was verified using an α-Syn PFF injection mouse model, and the results showed that ATC161 showed therapeutic effects in degrading brain α-Syn aggregates. AUTOTAC is the first technology to be shown to selectively degrade α-Syn aggregates in vitro and in vivo. In addition, oral administration of ATC161 has completed toxicology studies and is scheduled to enter phase I clinical studies in preparation for a phase II study in PD patients.

## 6. CMA-Dependent Autophagic Degradation Strategy

**Figure 11:**
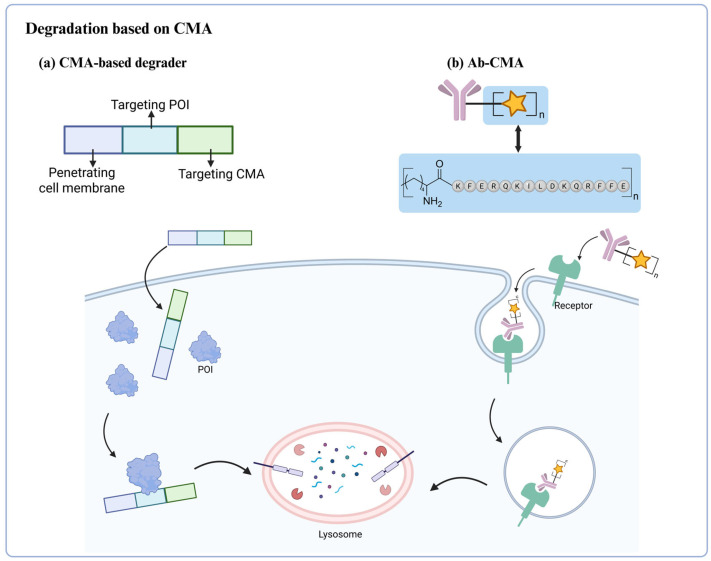
Schematic representation of CMA-based degrader and Ab-CMA for protein degradation. (**a**) The CMPD component of the degrader assists in transporting it across the cell membrane. Following intracellular entry, the PBD segment of the degrader specifically binds to the target protein. Subsequently, the CTM part of the degrader directs the target protein complex toward the lysosomal degradation pathway. (**b**) By conjugating a CMA-targeting sequence to an antibody, the complex is internalized upon target binding. After internalization, the CMA sequence directs the targeted protein complex to the lysosome via chaperone-mediated autophagy.

In addition to the previously discussed autophagy pathways, chaperone-mediated autophagy (CMA) has garnered significant attention in recent years, as a distinctive degradation mechanism. Consequently, there has been an increasing emphasis on the development of degradation technologies associated with this pathway. As early as 2014, Wang et al. engineered a targeting peptide that utilizes the CMA pathway to degrade endogenous proteins both in vitro and in vivo [64]. This degradation peptide consists of three components: the cell membrane penetration domain (CMPD) for crossing the blood-brain barrier, and the plasma membrane; protein-binding domains (PBDs) for selective binding to the POI; and the CMA-targeted motif (CTM) for directing complexes to the lysosomal degradation pathway (Figure 11a). As a proof of concept, the research team initially developed the targeting peptide TAT-GluN2B-CTM and demonstrated its selectivity for degrading Death-associated protein kinase 1 (DAPK1) [65]. To verify the general applicability of this platform, the team designed TAT-βSyn-CTM and TAT-GluN2B9c-CTM to further explore the degradation of two endogenous neuronal proteins, α-Syn and postsynaptic density protein 95 (PSD-95). The results showed that both peptides effectively facilitated the degradation of the two targets. This method can be easily generalized to potentially degrade any cytosolic POI, providing a new approach to regulate natural proteins.

Shao et al. in 2024 [66] designed an innovative protein degradation platform, based on antibody-peptide conjugates (Ab-CMAs), by strategically fusing commercial monoclonal antibodies with a chaperone-mediated autophagy (CMA)-targeting peptide (KFERQKILDQRFFE) to facilitate the specific degradation of cell-surface proteins via lysosomal pathways (Figure 11b) [66]. Mechanistic investigations have revealed that Ab-CMA facilitates the degradation of cell surface membrane proteins through the CMA pathway, critically dependent on the markers Hsc70 and LAMP2A, with its efficacy significantly diminished by lysosomal inhibitors, thereby confirming lysosome-dependent targeting via this pathway. The research team conducted a systematic validation of this platform through both in vitro and in vivo studies. In cellular models, cetuximab-conjugated Ctx-CMA demonstrated effective clearance of EGFR from the membranes of HeLa cells. Similarly, the constructs APL-CMA (targeting PDL1) and AH-CMA (targeting HER2) achieved comparable degradation of their respective targets in MDA-MB-231 and Huh7 cell lines. Notably, in A549 tumor-bearing BALB/c nude mice, intravenous administration of Ctx-CMA surpassed conventional cetuximab therapy, exhibiting both enhanced tumor growth suppression and a significant reduction in EGFR protein levels compared to control groups (IgG-CMA and Ctx alone). As a modular degradation strategy, Ab-CMA can effectively degrade various cell surface membrane proteins and has shown promise for in vivo therapeutic applications.

## 7. Autophagic Degradation Strategy Based on Nanotechnology

In recent years, the application of nanotechnology has gained significant popularity. This discipline presents numerous advantages, including the precise targeting of specific cells or tissues, facilitated by the modification of nanomaterials’ surface properties. The size, shape, and other characteristics of these nanomaterials can be meticulously controlled to fulfill specific application requirements. The incorporation of nanotechnology into biological research holds considerable potential, continuously broadening its research scope and diversifying its application scenarios [67].

**Figure 12:**
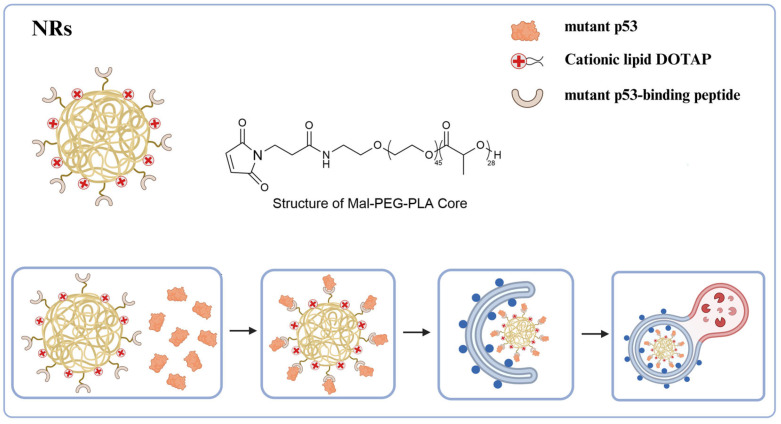
Schematic representation of nanoreceptor for protein degradation. On the NR surface, mutant p53-binding peptide specifically targets and binds to mutant p53. Concurrently, the cationic liposomal formulation DOTAP enhances autophagy level by inducing autophagosome formation. This process facilitates the delivery of NR-bound mutp53 into autophagosomes, thereby mediating its degradation.

P53 is a crucial tumor suppressor gene, and wild-type p53 plays a significant role in regulating the cell cycle and apoptosis [68,69,70]. Mutations in this gene occur in more than 50% of malignant tumors [71]. Mutant p53 (mutp53) increases the risk of cancer, subsequently promoting tumor development and metastasis. Among the various treatments for mutp53, the most direct approach is to induce its degradation. Huang et al. [72] have developed a multifunctional biomimetic nanoreceptor (NR) capable of concurrently binding to mutant p53 (mutp53), enhancing autophagy, and facilitating the degradation of mutp53. This NR is self-assembled from FDA-approved maleimide-polyethylene glycol-polylactic acid (Mal-PEG-PLA) nanoparticles (NPs), which contain 1, 2-dioleyl-3-trimethylammonium propane (DOTAP, a cationic lipid), and then covalently modified with mutp53-binding peptide (MBP) (Figure 12). The cationic lipid DOTAP promotes autophagosome formation, thereby augmenting autophagy levels and efficiently directing NR-bound mutant p53 proteins into the autophagic degradation pathway. These NRs demonstrate high selectivity in binding and degrading mutant proteins, enabling them to target various forms of mutp53 without affecting the anticancer wild-type p53 protein or the homologous p53 family member, p63. Following the degradation of mutp53 in tumor cells by NRs, the acquired functions of mutp53, such as the inhibition of cell proliferation, migration, and drug resistance, are mitigated, ultimately leading to the induction of cancer cell death. Additionally, NRs demonstrate excellent anti-tumor efficacy in the ES-2 (p53 S241F) ovarian cancer model and the human tumor xenograft PDX (p53 P72R^+/+^, C141Y^+/+^, and L350P^+/−^) ovarian cancer model, further underscoring their potential clinical application value. As a novel biomimetic nanoplatform, this nanotechnology can induce autophagy to target and degrade pathogenic proteins while efficiently delivering drugs. Its high selectivity minimizes safety risks associated with non-specific degradation, highlighting the broad clinical application prospects of NRs and providing a new direction for the precise treatment of major diseases such as cancer.

## 8. Autophagic Degradation Strategy Based on Limited Membrane Damage

**Figure 13:**
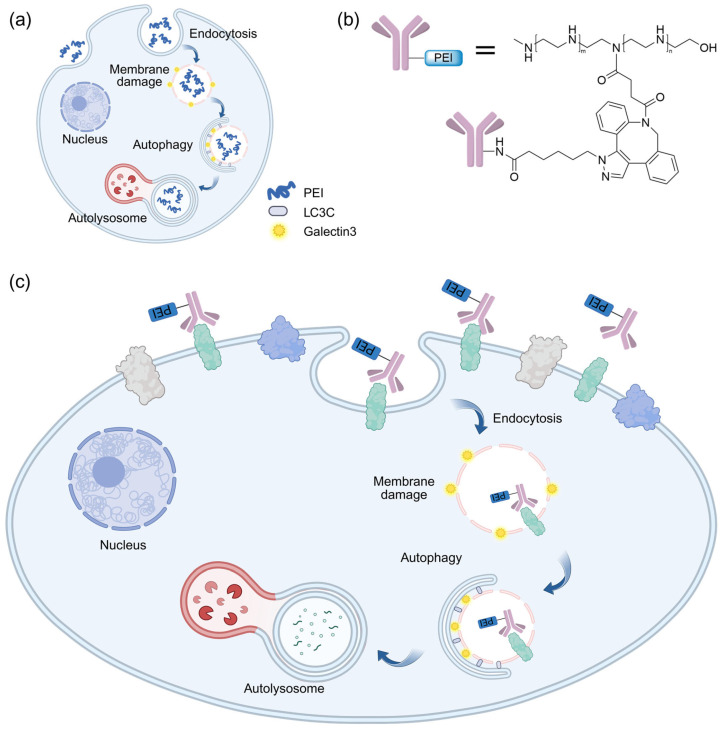
Schematic representation of AUTAB for protein degradation. (**a**) The mechanism of autophagy induced by PEI. Upon cellular internalization by endocytosis, PEI induces damage on the endosomal membrane, consequently leading to activation of the LC3C-mediated autophagy pathway and subsequent redirection of endosomes toward lysosomal degradation. (**b**) The chemical structure of AUTAB. (**c**) The mechanism of AUTAB for proteins degradation. Antibodies conjugated to PEI bind to target proteins and facilitate their endocytosis. Upon internalization, PEI induces endosomal membrane damage, thereby activating the LC3C-mediated autophagy pathway.

In addition to the autophagy-related degradation techniques above discussed, our team has recently developed a novel autophagy-based approach for the degradation of membrane proteins, referred to as AUTAB [73]. Distinct from these existing autophagy-related methodologies, AUTAB uniquely harnesses the receptor-independent autophagy pathway, a system that is inherently involved in membrane repair, to facilitate the targeted degradation of membrane proteins. Our investigations revealed that polyethylenimine (PEI), a cationic polymer commonly employed in gene and drug delivery [74,75], initiates a non-canonical LC3C-mediated autophagy by disrupting endosomal membrane integrity (Figure 13a). By leveraging this mechanism, we have engineered AUTAB by covalently conjugating PEI to the antibodies that specifically bind to cell membrane proteins (Figure 13b). Upon AUTAB binding to cell surface receptors via the antibody component, the PEI moiety induces localized endosomal disruption at the target site, thereby triggering LC3C-dependent autophagic flux and subsequent lysosomal degradation of the target membrane proteins (Figure 13c).

By conjugating PEI with therapeutic antibodies directed against diverse membrane proteins, the platform has effectively enabled the targeted degradation of clinically relevant receptors, including PD-L1, EGFR, and CD73. Remarkably, this degradation can be achieved at nanomolar concentrations and completed within 12 h across various cell types, underscoring the platform’s high degradation efficiency. In terms of the chemical mechanism, our findings indicate that the positive charge of PEI is critical for the activity of AUTAB. When the positive charge of PEI is obstructed, both its autophagy-inducing and degradation capabilities are significantly diminished. Additionally, AUTAB constructs derived from other positively charged polylysine derivatives exhibit the ability to induce the degradation of target proteins.

To improve the accessibility and convenience of AUTAB technology, we further developed a modular Nano-AUTAB system by covalently attaching PEI to the universal nano-secondary antibodies capable of recognizing IgG antibodies. This configuration facilitates effective collaboration with various antibodies specific to cell membrane proteins, enabling the autophagic degradation of a diverse array of these proteins. Consequently, compared to other technologies, this system exhibits high degradation efficiency, broad-spectrum applicability, and operational simplicity, thereby presenting substantial potential to advance fundamental biological research and therapeutic applications.

## 9. Conclusions

Targeted degradation technology compensates for the shortcomings of traditional small molecule inhibitors and is committed to solving the problem of undruggable targets. The current mainstream strategies utilize the ubiquitin-proteasome and endo-lysosomal pathways, and have derived popular technologies such as PROTAC and LYTAC. Leveraging autophagy for degradation presents a novel approach. Autophagy is a fundamental biological process in cells and is essential for maintaining the stability of the intracellular environment and the normal physiological functions of cells. The scope of autophagic degradation can cover many undruggable targets. This review describes related technologies, including ATTEC, AUTAC, AUTOTAC, CMA-based degradation, nanotechnology degradation strategy, and AUTAB. These technologies have demonstrated effective degradation of target proteins and organelles. While the potential of autophagy degradation technology is promising, challenges remain to be addressed.

First, specificity is the central challenge in targeted degradation technology. In the complex cellular environment, it is difficult to accurately target the desired protein and reduce the impact on non-target proteins, especially when targeting mutant proteins. The presence of off-target effects may lead to the degradation of other functional proteins, which in turn affects the normal function of the cell. Therefore, screening or designing specific target ligands is of main importance. Previous studies have revealed that autophagy is a complex and precise process involving the coordinated action of multiple proteins. The techniques described in this review all use autophagy to degrade the target substance. It is necessary to explore whether these techniques will affect the overall autophagy level of the cell. In addition, some substructures of the autophagy process, including non-target proteins, phospholipids, and organelles on autophagosomes and related endosomes, are theoretically degraded in lysosomes. The impact on these components still needs to be clarified. Quantitative proteomics can be used to analyze this specificity issue. This technique can precisely identify and quantify target proteins to determine the complete effect of a designed degrader on proteins. For example, it can be used to determine whether changes occur in autophagy-related substrate proteins, and whether a compound interacts with proteins beyond the intended target. In vitro pull-down experiment combined with immunoprecipitation assay can also help us determine whether the degrader interacts with proteins other than the designated target. Thus, it is possible for the researcher to determine whether the target protein is degraded and analyze off-target effects to reduce potential side effects.

Second, the mechanism of action of the technology described in this article still needs to be further investigated. For example, it is still necessary to conduct in-depth research on the mechanisms of ATTEC binding to LC3B and whether AUTAC entirely depends on the autophagy-lysosome pathway. The questions about the mechanisms of action can be solved with the help of structural and cell biology. We can utilize structural biology to apply physical methods for studying the structure, function, and interactions of biomolecules. Additionally, we can employ cell biology to investigate the molecular mechanisms of degradation processes.

Finally, there is a lack of systematic in vivo experimental assessment criteria for the aforementioned degradation techniques. Establishing a comprehensive standard would allow for the evaluation of in vivo effects, distribution, drug metabolism kinetics, and toxicity. While some techniques demonstrate clear effects in vitro, their in vivo efficacy remains uncertain, particularly concerning precise tissue distribution. Therefore, whether these techniques can achieve tissue-specific targeted degradation is an area that still requires exploration.

In future research, not only will the mechanism of autophagy induction and degradation need to be studied, but compounds will also need to be screened and designed to obtain particular molecules to optimize the autophagy degradation system. Artificial intelligence (AI) and machine learning (ML) have already been applied in various fields, and the trend of their intersection with biology is becoming increasingly apparent. In the future, AI/ML can be used to discover potential drug candidates for biological targets by performing large-scale computer simulations and virtual high-throughput screening of compound libraries, further optimizing the specificity and selectivity of the compounds for biological targets. In autophagy degradation technology, the advantages of AI/ML can be used to expand the compound libraries of L3CB ligands and p62 protein ligands. It can also be used to optimize existing compound molecules, such as further optimization of the S-guanylation tag in AUTAC molecules. In addition, AI/ML can be used to help predict the 3D structure of target proteins, provide information for chemical synthesis, and predict the possible effects of drug candidates on targets, including predicting affinity and potential toxicity.

As research advances, autophagy-based degradation technologies are poised to address challenges in treating a range of diseases, including but not limited to tumors, neurodegenerative disorders, autoimmune diseases, and bacterial and viral infections. Given the advantages of these technologies, we predict that it will also attract more and more institutions to establish and deploy patents in the future to accelerate the transformation of autophagic degradation technologies from laboratory research to clinical application. Currently, the Lu team of ATTEC technology and the Yong team of AUTOTAC have both established companies dedicated to transforming achievements and promoting the research and development of a new generation of degraders.

## References

[1] Dale B., Cheng M., Park K.S., Kaniskan H.U., Xiong Y., Jin J. (2021). Advancing targeted protein degradation for cancer therapy. Nat. Rev. Cancer.

[2] Zhang C., Liu Y., Li G., Yang Z., Han C., Sun X., Sheng C., Ding K., Rao Y. (2024). Targeting the undruggables-the power of protein degraders. Sci. Bull..

[3] Ding Y., Fei Y., Lu B. (2020). Emerging New Concepts of Degrader Technologies. Trends Pharmacol. Sci..

[4] Lu Y., Yang Y., Zhu G., Zeng H., Fan Y., Guo F., Xu D., Wang B., Chen D., Ge G. (2023). Emerging Pharmacotherapeutic Strategies to Overcome Undruggable Proteins in Cancer. Int. J. Biol. Sci..

[5] Li Y.Y., Yang Y., Zhang R.S., Ge R.X., Xie S.B. (2025). Targeted degradation of membrane and extracellular proteins with LYTACs. Acta Pharmacol. Sin..

[6] Wells J.A., Kumru K. (2024). Extracellular targeted protein degradation: An emerging modality for drug discovery. Nat. Rev. Drug Discov..

[7] Nalawansha D.A., Crews C.M. (2020). PROTACs: An Emerging Therapeutic Modality in Precision Medicine. Cell Chem. Biol..

[8] Bekes M., Langley D.R., Crews C.M. (2022). PROTAC targeted protein degraders: The past is prologue. Nat. Rev. Drug Discov..

[9] Banik S.M., Pedram K., Wisnovsky S., Ahn G., Riley N.M., Bertozzi C.R. (2020). Lysosome-targeting chimaeras for degradation of extracellular proteins. Nature.

[10] Pettersson M., Crews C.M. (2019). PROteolysis TArgeting Chimeras (PROTACs)—Past, present and future. Drug Discov. Today Technol..

[11] Zhao L., Zhao J., Zhong K., Tong A., Jia D. (2022). Targeted protein degradation: Mechanisms, strategies and application. Signal Transduct. Target. Ther..

[12] Debnath J., Gammoh N., Ryan K.M. (2023). Autophagy and autophagy-related pathways in cancer. Nat. Rev. Mol. Cell Biol..

[13] Cao W., Li J., Yang K., Cao D. (2021). An overview of autophagy: Mechanism, regulation and research progress. Bull. Cancer.

[14] Mizushima N., Yoshimori T., Levine B. (2010). Methods in mammalian autophagy research. Cell.

[15] Stavoe A.K.H., Holzbaur E.L.F. (2020). Neuronal autophagy declines substantially with age and is rescued by overexpression of WIPI2. Autophagy.

[16] Ghosh A.K., Mau T., O’Brien M., Garg S., Yung R. (2016). Impaired autophagy activity is linked to elevated ER-stress and inflammation in aging adipose tissue. Aging.

[17] Oshima M., Seki T., Kurauchi Y., Hisatsune A., Katsuki H. (2019). Reciprocal Regulation of Chaperone-Mediated Autophagy/Microautophagy and Exosome Release. Biol. Pharm. Bull..

[18] Kroemer G., Marino G., Levine B. (2010). Autophagy and the integrated stress response. Mol. Cell.

[19] Chang C., Jensen L.E., Hurley J.H. (2021). Autophagosome biogenesis comes out of the black box. Nat. Cell Biol..

[20] Melia T.J., Lystad A.H., Simonsen A. (2020). Autophagosome biogenesis: From membrane growth to closure. J. Cell Biol..

[21] Zhang Y., Liu X., Klionsky D.J., Lu B., Zhong Q. (2022). Manipulating autophagic degradation in human diseases: From mechanisms to interventions. Life Med..

[22] Xie Z., Klionsky D.J. (2007). Autophagosome formation: Core machinery and adaptations. Nat. Cell Biol..

[23] Lee Y.K., Lee J.A. (2016). Role of the mammalian ATG8/LC3 family in autophagy: Differential and compensatory roles in the spatiotemporal regulation of autophagy. BMB Rep..

[24] Mizushima N., Yoshimori T., Ohsumi Y. (2011). The role of Atg proteins in autophagosome formation. Annu. Rev. Cell Dev. Biol..

[25] Rogov V., Dotsch V., Johansen T., Kirkin V. (2014). Interactions between autophagy receptors and ubiquitin-like proteins form the molecular basis for selective autophagy. Mol. Cell.

[26] Nair U., Yen W.L., Mari M., Cao Y., Xie Z., Baba M., Reggiori F., Klionsky D.J. (2012). A role for Atg8-PE deconjugation in autophagosome biogenesis. Autophagy.

[27] Takahashi D., Arimoto H. (2021). Selective autophagy as the basis of autophagy-based degraders. Cell Chem. Biol..

[28] Ichimura Y., Kumanomidou T., Sou Y.S., Mizushima T., Ezaki J., Ueno T., Kominami E., Yamane T., Tanaka K., Komatsu M. (2008). Structural basis for sorting mechanism of p62 in selective autophagy. J. Biol. Chem..

[29] Marshall R.S., Hua Z., Mali S., McLoughlin F., Vierstra R.D. (2019). ATG8-Binding UIM Proteins Define a New Class of Autophagy Adaptors and Receptors. Cell.

[30] Kaushik S., Cuervo A.M. (2018). The coming of age of chaperone-mediated autophagy. Nat. Rev. Mol. Cell Biol..

[31] Ferreira J.V., Fofo H., Bejarano E., Bento C.F., Ramalho J.S., Girao H., Pereira P. (2013). STUB1/CHIP is required for HIF1A degradation by chaperone-mediated autophagy. Autophagy.

[32] Li Z., Wang C., Wang Z., Zhu C., Li J., Sha T., Ma L., Gao C., Yang Y., Sun Y. (2019). Allele-selective lowering of mutant HTT protein by HTT-LC3 linker compounds. Nature.

[33] Tong H., Yang T., Xu S., Li X., Liu L., Zhou G., Yang S., Yin S., Li X.J., Li S. (2024). Huntington’s Disease: Complex Pathogenesis and Therapeutic Strategies. Int. J. Mol. Sci..

[34] Zhao H.Y., Wang H.P., Mao Y.Z., Zhang H., Xin M., Xi X.X., Lei H., Mao S., Li D.H., Zhang S.Q. (2022). Discovery of Potent PROTACs Targeting EGFR Mutants through the Optimization of Covalent EGFR Ligands. J. Med. Chem..

[35] Lemmon M.A., Schlessinger J. (2010). Cell signaling by receptor tyrosine kinases. Cell.

[36] Zhu Z., Li J., Shen S., Al-Furas H., Li S., Tong Y., Li Y., Zeng Y., Feng Q., Chen K. (2024). Targeting EGFR degradation by autophagosome degraders. Eur. J. Med. Chem..

[37] Liao X., Qin G., Liu Z., Ren J., Qu X. (2024). Bioorthogonal Aptamer-ATTEC Conjugates for Degradation of Alpha-Synuclein via Autophagy-Lysosomal Pathway. Small.

[38] He H., Zhou C., Chen X. (2023). ATNC: Versatile Nanobody Chimeras for Autophagic Degradation of Intracellular Unligandable and Undruggable Proteins. J. Am. Chem. Soc..

[39] Chen H., Wang S., Zhang H., Nice E.C., Huang C. (2016). Nicotinamide phosphoribosyltransferase (Nampt) in carcinogenesis: New clinical opportunities. Expert Rev. Anticancer Ther..

[40] Korotchkina L., Kazyulkin D., Komarov P.G., Polinsky A., Andrianova E.L., Joshi S., Gupta M., Vujcic S., Kononov E., Toshkov I. (2020). OT-82, a novel anticancer drug candidate that targets the strong dependence of hematological malignancies on NAD biosynthesis. Leukemia.

[41] Dong G., Wu Y., Cheng J., Chen L., Liu R., Ding Y., Wu S., Ma J., Sheng C. (2022). Ispinesib as an Effective Warhead for the Design of Autophagosome-Tethering Chimeras: Discovery of Potent Degraders of Nicotinamide Phosphoribosyltransferase (NAMPT). J. Med. Chem..

[42] Bao J., Chen Z., Li Y., Chen L., Wang W., Sheng C., Dong G. (2024). Discovery of Novel PDEdelta Autophagic Degraders: A Case Study of Autophagy-Tethering Compound (ATTEC). ACS Med. Chem. Lett..

[43] Lu S., Jang H., Muratcioglu S., Gursoy A., Keskin O., Nussinov R., Zhang J. (2016). Ras Conformational Ensembles, Allostery, and Signaling. Chem. Rev..

[44] Schmick M., Vartak N., Papke B., Kovacevic M., Truxius D.C., Rossmannek L., Bastiaens P.I.H. (2014). KRas localizes to the plasma membrane by spatial cycles of solubilization, trapping and vesicular transport. Cell.

[45] Zimmermann G., Papke B., Ismail S., Vartak N., Chandra A., Hoffmann M., Hahn S.A., Triola G., Wittinghofer A., Bastiaens P.I. (2013). Small molecule inhibition of the KRAS-PDEdelta interaction impairs oncogenic KRAS signalling. Nature.

[46] Martin-Gago P., Fansa E.K., Klein C.H., Murarka S., Janning P., Schurmann M., Metz M., Ismail S., Schultz-Fademrecht C., Baumann M. (2017). A PDE6delta-KRas Inhibitor Chemotype with up to Seven H-Bonds and Picomolar Affinity that Prevents Efficient Inhibitor Release by Arl2. Angew. Chem. Int. Ed. Engl..

[47] Papke B., Murarka S., Vogel H.A., Martin-Gago P., Kovacevic M., Truxius D.C., Fansa E.K., Ismail S., Zimmermann G., Heinelt K. (2016). Identification of pyrazolopyridazinones as PDEdelta inhibitors. Nat. Commun..

[48] Onal G., Kutlu O., Gozuacik D., Dokmeci Emre S. (2017). Lipid Droplets in Health and Disease. Lipids Health Dis..

[49] Donnelly K.L., Smith C.I., Schwarzenberg S.J., Jessurun J., Boldt M.D., Parks E.J. (2005). Sources of fatty acids stored in liver and secreted via lipoproteins in patients with nonalcoholic fatty liver disease. J. Clin. Investig..

[50] Liu L., Zhang K., Sandoval H., Yamamoto S., Jaiswal M., Sanz E., Li Z., Hui J., Graham B.H., Quintana A. (2015). Glial lipid droplets and ROS induced by mitochondrial defects promote neurodegeneration. Cell.

[51] Sharma S., Adrogue J.V., Golfman L., Uray I., Lemm J., Youker K., Noon G.P., Frazier O.H., Taegtmeyer H. (2004). Intramyocardial lipid accumulation in the failing human heart resembles the lipotoxic rat heart. FASEB J..

[52] Fu Y., Chen N., Wang Z., Luo S., Ding Y., Lu B. (2021). Degradation of lipid droplets by chimeric autophagy-tethering compounds. Cell Res..

[53] Jia F., Wang X., Fu Y., Zhao S.M., Lu B., Wang C. (2024). DDHD2, whose mutations cause spastic paraplegia type 54, enhances lipophagy via engaging ATG8 family proteins. Cell Death Differ..

[54] Shribman S., Reid E., Crosby A.H., Houlden H., Warner T.T. (2019). Hereditary spastic paraplegia: From diagnosis to emerging therapeutic approaches. Lancet Neurol..

[55] Schuurs-Hoeijmakers J.H., Geraghty M.T., Kamsteeg E.J., Ben-Salem S., de Bot S.T., Nijhof B., van de Vondervoort I., van der Graaf M., Nobau A.C., Otte-Holler I. (2012). Mutations in DDHD2, encoding an intracellular phospholipase A(1), cause a recessive form of complex hereditary spastic paraplegia. Am. J. Hum. Genet..

[56] Tan S., Wang D., Fu Y., Zheng H., Liu Y., Lu B. (2023). Targeted clearance of mitochondria by an autophagy-tethering compound (ATTEC) and its potential therapeutic effects. Sci. Bull..

[57] Takahashi D., Moriyama J., Nakamura T., Miki E., Takahashi E., Sato A., Akaike T., Itto-Nakama K., Arimoto H. (2019). AUTACs: Cargo-Specific Degraders Using Selective Autophagy. Mol. Cell.

[58] Nakagawa I., Amano A., Mizushima N., Yamamoto A., Yamaguchi H., Kamimoto T., Nara A., Funao J., Nakata M., Tsuda K. (2004). Autophagy defends cells against invading group A Streptococcus. Science.

[59] Ito C., Saito Y., Nozawa T., Fujii S., Sawa T., Inoue H., Matsunaga T., Khan S., Akashi S., Hashimoto R. (2013). Endogenous nitrated nucleotide is a key mediator of autophagy and innate defense against bacteria. Mol. Cell.

[60] Takahashi D., Ora T., Sasaki S., Ishii N., Tanaka T., Matsuda T., Ikeda M., Moriyama J., Cho N., Nara H. (2023). Second-Generation AUTACs for Targeted Autophagic Degradation. J. Med. Chem..

[61] Ji C.H., Kim H.Y., Lee M.J., Heo A.J., Park D.Y., Lim S., Shin S., Ganipisetti S., Yang W.S., Jung C.A. (2022). The AUTOTAC chemical biology platform for targeted protein degradation via the autophagy-lysosome system. Nat. Commun..

[62] Lee J., Sung K.W., Bae E.J., Yoon D., Kim D., Lee J.S., Park D.H., Park D.Y., Mun S.R., Kwon S.C. (2023). Targeted degradation of ⍺-synuclein aggregates in Parkinson’s disease using the AUTOTAC technology. Mol. Neurodegener..

[63] Luk K.C., Kehm V., Carroll J., Zhang B., O’Brien P., Trojanowski J.Q., Lee V.M. (2012). Pathological alpha-synuclein transmission initiates Parkinson-like neurodegeneration in nontransgenic mice. Science.

[64] Fan X., Jin W.Y., Lu J., Wang J., Wang Y.T. (2014). Rapid and reversible knockdown of endogenous proteins by peptide-directed lysosomal degradation. Nat. Neurosci..

[65] Henshall D.C., Araki T., Schindler C.K., Shinoda S., Lan J.Q., Simon R.P. (2003). Expression of death-associated protein kinase and recruitment to the tumor necrosis factor signaling pathway following brief seizures. J. Neurochem..

[66] Shao J., Lin X., Wang H., Zhao C., Yao S.Q., Ge J., Zeng S., Qian L. (2024). Targeted Degradation of Cell-Surface Proteins via Chaperone-Mediated Autophagy by Using Peptide-Conjugated Antibodies. Angew. Chem. Int. Ed. Engl..

[67] Shi J., Kantoff P.W., Wooster R., Farokhzad O.C. (2017). Cancer nanomedicine: Progress, challenges and opportunities. Nat. Rev. Cancer.

[68] Shaw P.H. (1996). The role of p53 in cell cycle regulation. Pathol. Res. Pract..

[69] Vousden K.H., Lane D.P. (2007). p53 in health and disease. Nat. Rev. Mol. Cell Biol..

[70] Vousden K.H., Ryan K.M. (2009). p53 and metabolism. Nat. Rev. Cancer.

[71] Cancer Genome Atlas Research N. (2011). Integrated genomic analyses of ovarian carcinoma. Nature.

[72] Huang X., Cao Z., Qian J., Ding T., Wu Y., Zhang H., Zhong S., Wang X., Ren X., Zhang W. (2024). Nanoreceptors promote mutant p53 protein degradation by mimicking selective autophagy receptors. Nat. Nanotechnol..

[73] Cheng B., Li M., Zheng J., Liang J., Li Y., Liang R., Tian H., Zhou Z., Ding L., Ren J. (2025). Chemically engineered antibodies for autophagy-based receptor degradation. Nat. Chem. Biol..

[74] Gao X., Yao L., Song Q., Zhu L., Xia Z., Xia H., Jiang X., Chen J., Chen H. (2011). The association of autophagy with polyethylenimine-induced cytotoxicity in nephritic and hepatic cell lines. Biomaterials.

[75] Lin C.W., Jan M.S., Kuo J.H. (2014). Autophagy-related gene expression analysis of wild-type and atg5 gene knockout mouse embryonic fibroblast cells treated with polyethylenimine. Mol. Pharm..

